# Journey to Motherhood in the First Year After Child Birth 

**Published:** 2016-09

**Authors:** Nahid Javadifar, Fereshteh Majlesi, Alireza Nikbakht, Saharnaz Nedjat, Ali Montazeri

**Affiliations:** 1 Reproductive Health Promotion Research Center, Ahvaz Jundishapur University of Medical Sciences, Ahvaz, Iran; 2 Department of Health Services, School of Public Health, Tehran University of Medical Sciences, Tehran, Iran; 3 Nursing and Midwifery School, Tehran University of Medical Sciences, Tehran, Iran; 4 Department of Epidemiology and Biostatistics, School of Public Health, Knowledge Utilization Research Centre, Tehran University of Medical Sciences, Tehran, Iran; 5 Iranian Institute for Health Sciences Research, Tehran, Iran

**Keywords:** Motherhood, Child Birth

## Abstract

**Objective:** Child bearing is a period of psychological challenges that must be viewed in a social context. This study reports the maternal transition from the perspective of Iranian first-time mothers in the first year after childbirth.

**Materials and methods:** Qualitative method was chosen for explanation of mothers’ individual experiences of motherhood.26 first-time mothers (aged 18-35 years old with various socio-economic status) who had delivered between 0-1 year prior to the interviews participated in the study.Data were collected through in-depth semi-structured interviews and interview transcripts were analyzed using the constant comparison method.

**Results:** The core category was called "Regaining advanced balance." There were several themes within this category: "internal conflicts", "encounter and interaction" and "internalization". They felt unpreparedness, lack of control over their lives, incomplete maternal feelings and unstable relation to their husbands and others. Within the first postpartum days and weeks a sort of attachment develops between mother and child as the mother starts to attain a better understanding of maternal feelings; she begins to accept the child as an independent identity and reconstructs herself. As the attachment to child deepens, the mother feels control over the affairs. She realizes a kind of development and integration in herself which specifically stems from becoming a mother and attempts to strengthen family bonds.

**Conclusion:** Through the expression of new mothers’ experiences toward motherhood, healthcare providers can reach a better perception of the emotional and psychological changes as well as the various aspects of mothers’ acceptance of their maternal role and helps a better preparation and presentation of effective training programs for mothers and families.

## Introduction

Becoming a mother in all societies has been considered as the most crucial role for women. Although some women avoid childbearing or defer it, becoming a mother is mostly inevitable. The birth of the first child despite being a happy event, puts the mother into crisis and lots of stress and faces her with new roles and responsibilities (1, 2). The first year after childbirth is not only a vital period regarding the physical, emotional, and psychological development, but also a significant time for challenging first -time mothers’ capacities in adjusting to their maternal roles (3).

In the process of becoming a mother a woman goes through a period of change, instability (4, 5) and reorganization of life (6). This psychological change can be influenced by the individual condition of a woman, her outlooks and beliefs, her social and economical condition, her preparedness and knowledge of the situation as well as her psychological condition. The more mothers are developed in this regard, the better they can adopt their maternal role; this signifies that the experience of being a mother for the first time should be looked upon within the specific social context to which a woman belongs (7).

Due to the fact that the researches on transition to motherhood have been mostly carried out in western countries, this study aims to provide a better understanding of this process in Iranian first-time mothers while identifying the factors affecting maternal adjustment.

Since the purpose of this study is examining women’s experiences of first-time motherhood, understanding the process of becoming a mother and the correlations between this process and the factors affecting it as well as developing a theory in this regard, Grounded theory method serves the intention of this study best.

## Materials and methods

***Research Participants***

Among women attending to primary health care centres twenty six first-time mothers who came for routine postnatal care visits for their infant and met the inclusion criteria were selected as the participants of this study. The study inclusion criteria were as follows: first- time mothers, aged over 18, speaking Persian, full term and singleton delivery, no prior history of high-risk pregnancy, having healthy born babies aged o-1 year, no history of depression, no acute or chronic health disorder before and after childbirth, living with their husbands and belonging to various ethnic and demographic backgrounds.

***Data collection***

The data collection method in this research includes face- to-face, semi-structured, in-depth interviews with the participants which were conducted by the first author of this paper. As the participant mothers were selected in health care centers, the interview locations were decided upon by them beforehand. During interviews they were asked to describe their postpartum experiences. Then the questions turned more specialized based on the concepts which arose from data analysis as well as those emerged through the construction and development of the theory. The number of interviews varied between one to two or even three times in some cases, depending on necessity or to clarify the collected data. The interviews were held individually and privately while the participants were assured of confidentiality of their information and were given the opportunity to freely discontinue their involvement at any time during the study .

In the beginning of each interview the participants were informed of the purpose of the research project and the interviews were recorded and transcribed verbatim, immediately after obtaining their permission.

Field notes were also taken to describe the concepts that appeared during the interviews, the observations, the setting as well as the participants’ condition. Interviews took thirty to sixty minutes per participant, depending on their conditions.

***Data Analysis***

In qualitative research, analysis takes place at the same time as data collection. The subsequent interviews, in this way, will be carried out in the right path of the process.

The data coding technique in this study is based on Strauss and Corbin’s (1998) (8) approch. First the interview transcripts and the key sentences were coded and the similar codes were arranged into major groups. These codes and clusters were compared again and the notes and transcribed interviews were reviewed to discern and blend the elements that were alike and then the related categories were lined up together; thus, the accuracy of the emerging themes was verified. The interviews were held untill data saturation was reached and no more new theme was evident in the collected data. After that the selective coding process was performed as the core variable was identified, the relevant categories were unified into a central category which represented the major phenomenon of the study. 

Reminders and notes were written down through the process of research while the codings and categories were being reviewed by the second and the third authors independently until the emerging themes were agreed upon.

***Procedures of the study***

After obtaining permission from the ethics committee of the Tehran University of Medical Sceiences, twenty six mothers who met the inclusion criteria for the study were selected from among those visiting the health care centers in Tehran and Ahwaz. Interviews were then conducted, recorded, and transcribed after informing the participants of the objectives of the study, receiving their written consent and determining the location and the time of interviews according to their wish.

## Results

The averege age of the participants was twenty six years old and the average length of their marriage at the time of interview was three years. Four of the participants were working women and the rest were housewives. Their educational level ranged from high school to master’s degree. Two of the participants were living with their husband’s family, one of them lived with her own family, and the rest had independent hoseholds. 

***Central Theme***

The central theme appearing from data analysis in this study was called “Regaining advanced balance” and the three sub- themes were considered as: internal conflicts, confrontation and interaction, and internalization. Although the phenomenon of transition to motherhood is believed to begin with pregnancy, the focus of the present study is on mothers’ postpartum experiences.

***Central Theme: Regaining advanced balance***

The analysis of the interviews and mothers’ experiences concerning maternal role attainment revealed that women in their confrontation with the crisis situation of childbearing constantly attempt to adapt to their maternal role so as to reach a positive balance toward the internalization of this role. The occurance of internal conflicts, confrontation and interaction, and internalization signifies a trasition from a state of disorder to order and balance, and acquisition of a new identity as well as a rite of passage from womanhood to motherhood which involves great changes.

***First Theme: Internal Conflicts***

The scrutiny of the mothers’ experiences in this study exhibits that during the first days and weeks postpartum mother experiences contradictory emotional states which indicate her incapability of perceiving her current situation and circumstances, and her new maternal role. She feels like she is not prepared enough to face the new situation as a mother and feels confused and bewildered, constantly thinking that her current status is inconsistent with her former expectations and the world she used to imagine about the child’s appearance and her life with a child. Fitting into her new role confronts the mother with new expectations which can, in turn, contribute to this feeling of unpreparedness. Not only does she set herself up with new expectations as a mother, but suddenly comes up against others’ expectations of herself for which she is not ready.

Feeling of inadequacy in controlling affairs especially during the first days and weeks after birth is outstanding among mothers’ experiences. The mother who used to live her normal life before childbirth, suddenly feels a loss of control over her life affairs and therefore develops a deep sense of inconvenience and trouble accompanied by a feeling of incapacity which derives from lack of self-assurance. Fear and anxiety about her child’s health and her future, etc. on the one hand, and concerns about the inefficiency of her feelings and attitudes toward the child on the other hand, add to this lack of mastery over life affairs. New moms experience a sense of neediness more than any other time. This feeling of neediness especially a desire to receive social, emotional, and practical supports is another indication of decreased control over the new circumstances.

*“Early after birth, as my baby cried I also used to cry with her. I just wanted to make her sleep. I thought I was unable do anything and was always concerned that I wouldn’t be able to raiseher... I found my baby bothersome and felt burdened by her.”*

Some mothers expressed that despite what they had heard or imagined about motherly affections, they didn’t feel that much affection toward their child and just tried to impose it on themselves, and thus felt guilty in this regard. 

*“I’ve not revealed it to anyone before, but I didn’t like her much at first; perhaps because I was really annoyed. I mean, I liked her but not as much as I should. I don’t know why (uttering these words in shame)"*

As the mother comes up with the new situation and directs all her attention toward the baby, marital relationships with her husband deteriorate and their verbal and nonverbal interactions as well as affectionate behaviours get restricted. She is concious and concerned about this instability of marital relations and strives to mend it despite all the existing pressures. Additionally, doing baby chores doesn’t leave her any time to have social interactions or leisure activities and this intensifies mother’s internal conflicts.

***Second Theme: Confrontation and interaction***

Mothers’ experiences indicate that mother- child emotional relationship develops gradually after birth. Mothers’ accounts of the first postnatal year shows that infants’ attachment behavior toward their mothers is associated with physical needs (i.e. nutrition, protection, etc.) rather than emotional needs; this also holds true with mothers’ attachment to the child which makes them responsive to the baby’s needs. However, this physical dependence gradually turns into emotional connection between infant and mother. Breastfeeding enhances the establishment of a deep and abiding relation between mother and child. Mother beginns to come up with a perception of mothehood and she learns that her feelings toward the baby is more than loving her. The participants declared that the way they loved their babies is incomparable to any other kind of love and affection they had previously experienced. These are the thoughts that occasionally happen to a new mom and involve her mind. 

*“I feel that it is not just the mother who loves the baby; the baby also develops affection for the mother. When you see that the baby is most comfortable when she finds you close to her and waits to receive your cuddles and hugs you feel, in turn, more attached to her.”*

The reciprocal responsiveness that occurs between mother and infant and mother’s recognition of baby as a distinct identity who is capable to respond to her emotional needs, helps her to have a better perception of the child and accept it as a separate identity.

Caring for the baby provides mother with a better perception of the child and makes her more attentive to the baby’s charactristics and traits and the way she expresses her needs; it also assists the process of accepting the child as an individual identity.

*“Since my baby has grown a little bit I feel like he is a grown up human being sitting beside me; a human being who cannot just speak; I feel that he can understand every thing. I talk to him a lot; I think he can understand every thing I say. I like him to call me mama as soon as possible. The more I see his reactions, the more I feel affectionate toward him. I was inclined to his initially but I didn’t know what it means to have a child, but as he smiles more I feel more attached to him and become more interested in him.”*

The analysis of the first-time mothers’ experiences in their maternal adaptation reveals the impact of mothers’ understanding of the current situation in adjusting themselves to their maternal role. It is apparent that perceiving the situation which results from mothers’ acceptance of the current condition, examination of their weak points and shortcomings, and their tendency to eliminate the problems related to taking on the mothering role, is an important element and the starting point in accepting the role of a mother and coping with its related issues. As mother begins to get along with the new circumstances, she tries to recognize her deficiencies in performing her maternal role.

*“I was alone with my baby, in such a chaotic situation. I found myself wasting a lot of time. I just wandered around at home and couldn’t do anything while all the house chores were left undone.”*

In order to have a better maternal role performance the mother should be able to restore her mental composure which can be reached by recognition of her status as a mother, wife, etc, and reorganization of her mental framework.

***Third Theme: Internalization***

As the baby grows a stronger and enduring attachment develops between mother and infant. The constancy of this attachment which takes place over time is rendered through such expressions as being unable to stay away from the child, or getting more interested in her or him, etc. The baby turns to be the most precious thing to the mother so that she wants everything including herself for the baby and puts her maternal duties above everything else. Thus the baby takes precedence in her life and she devotes herself to her needs. The stabilization and enhancement of maternal feelings occur simultaneously while the mother gains a more comprehensive understanding of motherhood. It was frequently heard in their speeches that “*now I can understand how my mother felt toward me”*

As the baby grows maternal behaviours and interactions with her change while motherly feelings remain unaffected in nature.

*“Motherly feelings remain always the same and just change in form and appearance. Once you are concerned about the baby’s crying and breastfeeding her. As she grows older you think about her teething, then walking, and then school, university, job, marriage…. When she is a child you kiss her and hug her; as she grows up, the ways of conveying maternal affections vary while the core feelings remain constant.”*

Mother’s competence and her capability in performing her role as she fulfils the needs of the child and those of the family and herself is indicative of the stability of maternal role and mother’s adjustment to it. Mothers’ contentment with the situation which is expressed through their description of life with a child as good and satisfactory suggests their dominance and control over affairs. Improvement of the child’s temperament, enhancement of mother’s physical and emotional state, and diminution of her fears and concerns about the child’s health which is brought about as the child grows, are all influential in developing a sense of satisfaction with the current situation. Maternal role satisfaction is probably the most notable indicator of contentment with conditions and mastery over affairs. 

Gaining competence is an important sign of mastery over life affairs. In order to attain competence mothers should be able to find the existing strategies and select the proper one(s) to solve the problems. Therefore, she should learn more about the baby’s characteristics and behaviours and the way the baby expresses her needs, and utilize her own potentials such as her planning ability, etc. to exert a better control over her life circumstances. Acquisition of competence and capability, independence of performance, and an increased pace of activities which is gained through persistent practice takes an important part in achieving control or as mothers say “getting into a regular life routine". Mothers, who received a long-term support especially from their mothers and families, had more problems in doing their maternal duties. Gaining a sense of merit and capability is a sign of mother’s dominance and control.

One of the changes that was unanimously indicated by the first-time mothers participating in the study is the occurrence of positive emotional changes, including a sense of being compassionate and getting less self-centered as well as an increased sense of responsibility ,hope and motivation. Social growth and advancement was another event that was almost mentioned by all mothers in expressing their feelings. They believed that they turned more considerate toward others especially children and mothers. They also had a greater appreciation for their parents, especially their mothers.

Mothers’ inclination toward making up social communications, especially with other mothers with whom they can discuss subjects related to children is another social development point out by the participants. Improved sense of self-adaptation and flexibility is another significant social change that was indicated by some of them. Increased ability to adjust to difficult situations and application of previously held learning and information was also mentioned in their accounts. Mothers also come up with a better understanding of their social roles and consciously attempt to differentiate between these roles and fulfill the requirements for each.

Feeling like a truly grown up was also shared by all mother participants. This kind of feeling is not due to being aroused or receiving more respect and social acceptability as a mother but stems from their behavioral changes and their new way of looking at matters. 

Stability of family bonds is another event that reveals itself over time through the strengthening of marital relations. By the internalization of maternal role and other aspects of maternal adaptation (as mentioned earlier) the conjugal relationship between husband and wife improves and returns to its normal state. The participants also reported indications of a development in the mutual expressions of affection with their husbands and a warmer and more intimate association between them which signifies an improvement of emotional attachments between couples.

By the consolidation of marital relations, the family concepts and values promote as well. Increased mutual trust ,the warmth and joy brought about for the couple, and the construction and reinforcement of the concept of” family” which takes place as the two- membered family changes into a three-membered one, are all influential in strengthening the family bonds. 

*“My husband and I didn’t use to trust each other and he refused to tell me many things before; he was closer to his own family than to me but since we have got a child we trust each other more than before… we feel more joy in our life. I am also more busy and entertained by the baby and life is less monotonous as if there was a gap which is now filled with our child.”*

Increased attachment of mother to her family life and her being more attentive and thoughtful in making decisions are the signs of family stability.

## Discussion

Studies show that the process of becoming a mother begins with pregnancy and maternal role attainments as well as maternal identity acquisition continue for several months after child birth (7). While lots of studies have been undertaken on transition to motherhood, less has been done on the process of maternal role attainment in the first year postpartum. 

Regaining advanced balance reveals that the changes that occur in women distort and then reconstruct the concept of “self” in them and therefore bring about new conditions which are accomponied with a sort of development and evolusion. All these changes eventually result in a new perception of the concepts of affection, sacrifice, and competence and induce a feeling of social -emotional development added to a sense of capability and acceptability in mothers. The results of other studies in this regard also depict that becoming a mother is accompanied with a change in the meaning of “self"(9) and also an individual growth and development (10).

 The postpartum internal conflicts decrease mother’s capabilities in adapting to her new role and disrupt her social and emotional well-being. Unpreparedness, feeling inadequate to control the situation, incomplete maternal emotions, and instability of relations are the factors that can provoke a sense of imbalance, and distort mother’s former perception of her “self” concept. Due to these complications the first days and weeks after giving birth turn into a hard time for the mother as Nystrom and Ohrling (2004 ) (11) consider the first year postpartum as an extra overwhelming period for parents to encounter.

According to Darvill (2010) (9), mother’s diminished perception of her “ self” initiates as the pregnancy is confirmed and continues till the time around delivery and from then, increases till giving birth and then decreases again after child birth.

The present study also acknowldges a decrease in mother’s perception of “self” and the distortion of her former balance which results from the occurance of drastic changes in mother’s condition.

Confrontation and engagement between mother and child creates a sense of attachment and leads to the perception and experience of motherly feelings. Mother’s concious reaction to the signals and actions that the baby takes on, is associated with her perception of the baby and their mutual interaction. Mother’s sensetivity to her baby’s affective signals intensifies mother-infant bond (12). Mother needs to harmonize herself with her new family form and she can acquire her maternal identity by taking care of the baby and setting up a relation with her (13).

Meanwhile mother’s perception of her child as a separate identity and her understanding of the circumstances as she regains her mental composure are the first signals of life balance reconstruction and modification of mother’s disrupted sense of “self”.

Although no specific time or order can be considered for the aforementioned occurrences that new mothers encounter, the findings of this research project are consistent with those of the other studies carried out in this regard.

It is said that mothers’ emotional state alters according to the child’s evolutionary stage (14). Mother’s attachment to her child strengthens and stabilizes and she gradually regards herself as belonging to the child and devotes herself to him (15); the child turns to be the center of attention and the parents’ first priority (16).

According to Yerkes-Dodson law the decrease or increase of arousal at very high levels result in a decrease in performance while the average level of arousal promotes an increase in task performance (17). Therefore, as the physical- emotional changes caused by pregnancy turn to the normal state, the baby moves from the stage of infancy, motherly fears and concerns about the child’s health and her survival are modified, and her performance of maternal duties improves, mother gets mastery over affairs and attains a sense of self- contentment.

As the mother feels easy about doing baby caring chores and is able to decode the child’s behavioral signals, she is supposed to have acquired maternal role competence. This sense of capability promotes maternal identity and fosters maternal role attainment (6, 18) Maternal role competence is affected by mother’s self-confidence, her sense of mastery in control of affairs, and the extent to which she feels attached to the child (19). Studies have also revealed that this sense of mastery over lifeaffairs is a strong predictor of health condition and family performance in postpartum period (20). Most of the investigations focus on the concept of motherhood during the first days and weeks after childbirth while less studies have been undertaken on mothers’ experiences during the first year postpartum.

Social-emotional development is defined as the process of effective application of knowledge, views, and necessary skills for recognition and control of emotions, as well as being more attentive toward others, making wise and responsible decisions, establishing positive mutual relationships, and ability to take control of challenging situations and adapt to them (21).The concept of development in the experiences of the participants in this study refers to both the improvement of their social and emotional functioning, and also the enhancement of their perceived self-image through which they felt even a change in their appearance, thinking that “they have grown up and look like mothers”; this proves the depth of the internal changes that mothers undergo and the emergence of a new definition of “self” which is the outcome of maternal role adaptation. This new insight of “self” which contains an integrated meaning of adaptation, can be considered as an important component in the examination of maternal role adaptation.

Studies have revealed that when conjugal relations are based on shared affection and mutual understanding and compassion between the spouses, women tend to be healthier and happier as they become mothers (22). The results of this study illustrate that instability of marital relationships which contributes to mother’s internal struggles during the first weeks after birth, shifts into a state of stability, and the family foundation strengthens as maternal identity is established and mother gains dominance over affairs, and thus performs a better maternal role. Therefore, it can be inferred from the participants’ experiences that improvement of marital relationships is another indication of a proper maternal role attainment.

## Conclusion

This study proceeds to explain both negative and positive aspects of maternal adaptation and mother’s psychological health as well as the specific consequences of maternal role adjustment, including family development and stabilization which differentiates this research from the previous studies that discuss the process of maternal adaptation under the category of stress (23). The researcher believes that this approach enables us to get a multi -dimensional picture of mothers’ experiences concerning maternal adjustment and the factors affecting it. Since in Iran as in many other countries prenatal courses and childbirth education programs are cantered on physical health issues rather than mental and psychological problems and the healthcare providers either have scanty information of parenting and maternal adaptation or do not give precedence to these subjects in their programs, the findings of this study can be applied to improve the maternal and child health programs and services.
